# Supplementary material for: Social Determinants of Health and 30-Day Readmission for Heart Failure Patients in U.S. Hospitals: Evidence from ICD-10 Z-Code Data
Source: Healthcare (Basel). 2025 Aug 23;13(17):2102. doi: 10.3390/healthcare13172102 (PMC12428219; doi:10.3390/healthcare13172102)
Supplement: Supplementary file 1 [file healthcare-13-02102-s001.zip › healthcare-3763537-supplementary.pdf]

Supplemental Materials

Table S1: ICD-10 Codes for Heart Failure

ICD-10 Code	ICD-10 Code Description
I0981	RHEUMATIC HEART FAILURE
I110	HYPERTENSIVE HEART DISEASE WITH HEART FAILURE
I130	HYP HRT & CHR KDNY DIS W HRT FAIL AND STG 1-4/UNSP CHR KDNY
I132	HYP HRT & CHR KDNY DIS W HRT FAIL AND W STG 5 CHR KDNY/ESRD
I42.xx	CARDIOMYOPATHY
I43	CARDIOMYOPATHY IN DISEASES CLASSIFIED ELSEWHERE
I50.xx	HEART FAILURE
I9713	POSTPROCEDURAL HEART FAILURE
O903	PERIPARTUM CARDIOMYOPATHY

Figure S1: Patient selection attrition chart

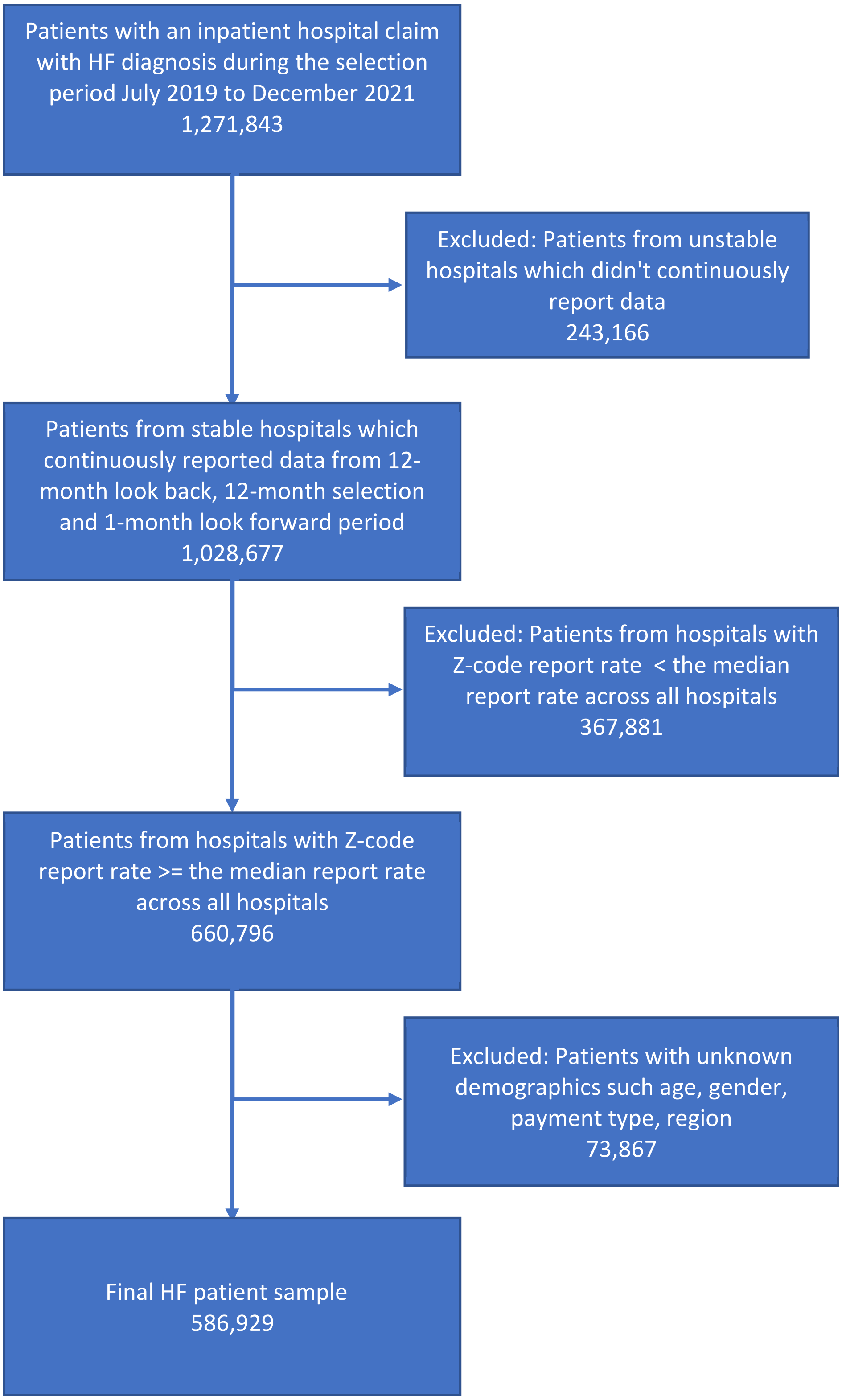

Table S2: ICD-10 Codes for Social Determinants of Health

SDoH Group	ICD-10 Code	ICD-10 Code Description
Z55: Education and literacy	Z55	PROBLEMS RELATED TO EDUCATION AND LITERACY
	Z55.0	ILLITERACY AND LOW-LEVEL LITERACY
	Z55.1	SCHOOLING UNAVAILABLE AND UNATTAINABLE
	Z55.2	FAILED SCHOOL EXAMINATIONS
	Z55.3	UNDERACHIEVEMENT IN SCHOOL
	Z55.4	EDUCATIONAL MALADJUSTMENT & DISCORD W TEACHERS & CLASSMATES
	Z55.8	OTHER PROBLEMS RELATED TO EDUCATION AND LITERACY
	Z55.9	PROBLEMS RELATED TO EDUCATION AND LITERACY, UNSPECIFIED
Z56: Employment	Z56	PROBLEMS RELATED TO EMPLOYMENT AND UNEMPLOYMENT
	Z56.0	UNEMPLOYMENT, UNSPECIFIED
	Z56.1	CHANGE OF JOB
	Z56.2	THREAT OF JOB LOSS
	Z56.3	STRESSFUL WORK SCHEDULE
	Z56.4	DISCORD WITH BOSS AND WORKMATES
	Z56.5	UNCONGENIAL WORK ENVIRONMENT
	Z56.6	OTHER PHYSICAL AND MENTAL STRAIN RELATED TO WORK
	Z56.8	OTHER PROBLEMS RELATED TO EMPLOYMENT
	Z56.81	SEXUAL HARASSMENT ON THE JOB
	Z56.82	MILITARY DEPLOYMENT STATUS
	Z56.89	OTHER PROBLEMS RELATED TO EMPLOYMENT
	Z56.9	UNSPECIFIED PROBLEMS RELATED TO EMPLOYMENT
Z57: Occupational exposure risk	Z57	OCCUPATIONAL EXPOSURE TO RISK FACTORS
	Z57.0	OCCUPATIONAL EXPOSURE TO NOISE
	Z57.1	OCCUPATIONAL EXPOSURE TO RADIATION
	Z57.2	OCCUPATIONAL EXPOSURE TO DUST
	Z57.3	OCCUPATIONAL EXPOSURE TO OTHER AIR CONTAMINANTS
	Z57.31	OCCUPATIONAL EXPOSURE TO ENVIRONMENTAL TOBACCO SMOKE
	Z57.39	OCCUPATIONAL EXPOSURE TO OTHER AIR CONTAMINANTS
	Z57.4	OCCUPATIONAL EXPOSURE TO TOXIC AGENTS IN AGRICULTURE
	Z57.5	OCCUPATIONAL EXPOSURE TO TOXIC AGENTS IN OTHER INDUSTRIES
	Z57.6	OCCUPATIONAL EXPOSURE TO EXTREME TEMPERATURE
	Z57.7	OCCUPATIONAL EXPOSURE TO VIBRATION
	Z57.8	OCCUPATIONAL EXPOSURE TO OTHER RISK FACTORS
	Z57.9	OCCUPATIONAL EXPOSURE TO UNSPECIFIED RISK FACTOR
Z59: Housing and economic	Z59	PROBLEMS RELATED TO HOUSING AND ECONOMIC CIRCUMSTANCES
	Z59.0	HOMELESSNESS
	Z59.1	INADEQUATE HOUSING
	Z59.2	DISCORD WITH NEIGHBORS, LODGERS AND LANDLORD
	Z59.3	PROBLEMS RELATED TO LIVING IN RESIDENTIAL INSTITUTION

	Z59.4	LACK OF ADEQUATE FOOD AND SAFE DRINKING WATER
	Z59.5	EXTREME POVERTY
	Z59.6	LOW INCOME
	Z59.7	INSUFFICIENT SOCIAL INSURANCE AND WELFARE SUPPORT
	Z59.8	OTHER PROBLEMS RELATED TO HOUSING AND ECONOMIC CIRCUMSTANCES
	Z59.9	PROBLEM RELATED TO HOUSING AND ECONOMIC CIRCUMSTANCES, UNSP
Z60: Social environment	Z60	PROBLEMS RELATED TO SOCIAL ENVIRONMENT
	Z60.0	PROBLEMS OF ADJUSTMENT TO LIFE-CYCLE TRANSITIONS
	Z60.2	PROBLEMS RELATED TO LIVING ALONE
	Z60.3	ACCULTURATION DIFFICULTY
	Z60.4	SOCIAL EXCLUSION AND REJECTION
	Z60.5	TARGET OF (PERCEIVED) ADVERSE DISCRIMINATION AND PERSECUTION
	Z60.8	OTHER PROBLEMS RELATED TO SOCIAL ENVIRONMENT
	Z60.9	PROBLEM RELATED TO SOCIAL ENVIRONMENT, UNSPECIFIED
Z62: Upbringing	Z62	PROBLEMS RELATED TO UPBRINGING
	Z62.0	INADEQUATE PARENTAL SUPERVISION AND CONTROL
	Z62.1	PARENTAL OVERPROTECTION
	Z62.2	UPBRINGING AWAY FROM PARENTS
	Z62.21	CHILD IN WELFARE CUSTODY
	Z62.22	INSTITUTIONAL UPBRINGING
	Z62.29	OTHER UPBRINGING AWAY FROM PARENTS
	Z62.3	HOSTILITY TOWARDS AND SCAPEGOATING OF CHILD
	Z62.6	INAPPROPRIATE (EXCESSIVE) PARENTAL PRESSURE
	Z62.8	OTHER SPECIFIED PROBLEMS RELATED TO UPBRINGING
	Z62.81	PERSONAL HISTORY OF ABUSE IN CHILDHOOD
	Z62.810	PERSONAL HISTORY OF PHYSICAL AND SEXUAL ABUSE IN CHILDHOOD
	Z62.811	PERSONAL HISTORY OF PSYCHOLOGICAL ABUSE IN CHILDHOOD
	Z62.812	PERSONAL HISTORY OF NEGLECT IN CHILDHOOD
	Z62.813	PERS HX OF FORCED LABOR OR SEXUAL EXPLOITATION IN CHILDHOOD
	Z62.819	PERSONAL HISTORY OF UNSPECIFIED ABUSE IN CHILDHOOD
	Z62.82	PARENT-CHILD CONFLICT
	Z62.820	PARENT-BIOLOGICAL CHILD CONFLICT
	Z62.821	PARENT-ADOPTED CHILD CONFLICT
	Z62.822	PARENT-FOSTER CHILD CONFLICT
	Z62.89	OTHER SPECIFIED PROBLEMS RELATED TO UPBRINGING
	Z62.890	PARENT-CHILD ESTRANGEMENT NEC
	Z62.891	SIBLING RIVALRY
	Z62.898	OTHER SPECIFIED PROBLEMS RELATED TO UPBRINGING
	Z62.9	PROBLEM RELATED TO UPBRINGING, UNSPECIFIED
Z63: Primary support group	Z63	OTH PROB REL TO PRIM SUPPORT GROUP, INC FAMILY CIRCUMSTANCES
	Z63.0	PROBLEMS IN RELATIONSHIP WITH SPOUSE OR PARTNER
	Z63.1	PROBLEMS IN RELATIONSHIP WITH IN-LAWS

	Z63.3	ABSENCE OF FAMILY MEMBER
	Z63.31	ABSENCE OF FAMILY MEMBER DUE TO MILITARY DEPLOYMENT
	Z63.32	OTHER ABSENCE OF FAMILY MEMBER
	Z63.4	DISAPPEARANCE AND DEATH OF FAMILY MEMBER
	Z63.5	DISRUPTION OF FAMILY BY SEPARATION AND DIVORCE
	Z63.6	DEPENDENT RELATIVE NEEDING CARE AT HOME
	Z63.7	OTHER STRESSFUL LIFE EVENTS AFFECTING FAMILY AND HOUSEHOLD
	Z63.71	STRESS ON FAM D/T RETURN OF FAMILY MEMBER FROM MILTRY DEPLOY
	Z63.72	ALCOHOLISM AND DRUG ADDICTION IN FAMILY
	Z63.79	OTHER STRESSFUL LIFE EVENTS AFFECTING FAMILY AND HOUSEHOLD
	Z63.8	OTHER SPECIFIED PROBLEMS RELATED TO PRIMARY SUPPORT GROUP
	Z63.9	PROBLEM RELATED TO PRIMARY SUPPORT GROUP, UNSPECIFIED
Z64-Z65: Psychosocial	Z64	PROBLEMS RELATED TO CERTAIN PSYCHOSOCIAL CIRCUMSTANCES
	Z64.0	PROBLEMS RELATED TO UNWANTED PREGNANCY
	Z64.1	PROBLEMS RELATED TO MULTIPARITY
	Z64.4	DISCORD WITH COUNSELORS
	Z65	PROBLEMS RELATED TO OTHER PSYCHOSOCIAL CIRCUMSTANCES
	Z65.0	CONVICTION IN CIVIL & CRIMINAL PROCEEDINGS W/O IMPRISONMENT
	Z65.1	IMPRISONMENT AND OTHER INCARCERATION
	Z65.2	PROBLEMS RELATED TO RELEASE FROM PRISON
	Z65.3	PROBLEMS RELATED TO OTHER LEGAL CIRCUMSTANCES
	Z65.4	VICTIM OF CRIME AND TERRORISM
	Z65.5	EXPOSURE TO DISASTER, WAR AND OTHER HOSTILITIES
	Z65.8	OTH PROBLEMS RELATED TO PSYCHOSOCIAL CIRCUMSTANCES
	Z65.9	PROBLEM RELATED TO UNSPECIFIED PSYCHOSOCIAL CIRCUMSTANCES
Z77.1: Environmental pollution	Z77.1	CONTACT W AND EXPOSURE POLLUTN & HAZRD IN PHYS ENVR
	Z77.11	CONTACT W AND (SUSPECTED) EXPOSURE TO ENVIRON POLLUTION
	Z77.110	CONTACT WITH AND (SUSPECTED) EXPOSURE TO AIR POLLUTION
	Z77.111	CONTACT WITH AND (SUSPECTED) EXPOSURE TO WATER POLLUTION
	Z77.112	CONTACT WITH AND (SUSPECTED) EXPOSURE TO SOIL POLLUTION
	Z77.118	CONTACT W AND (SUSPECTED) EXPOSURE TO OTH ENVIRON POLLUTION
	Z77.12	CONTACT W AND EXPSR TO HAZARDS IN THE PHYSICAL ENVIRONMENT
	Z77.120	CONTACT WITH AND (SUSPECTED) EXPOSURE TO MOLD (TOXIC)
	Z77.121	CONTACT W AND EXPOSURE TO HARMFUL ALGAE AND ALGAE TOXINS
	Z77.122	CONTACT WITH AND (SUSPECTED) EXPOSURE TO NOISE
	Z77.123	CNTCT W & EXPSR TO RADON AND OTHER NATURALLY OCCUR RADIATION
	Z77.128	CONTACT W AND EXPSR TO OTH HAZARDS IN THE PHYSCL ENVIRONMENT
Z91.89: Transportation	Z91.89	OTH PERSONAL RISK FACTORS, NOT ELSEWHERE CLASSIFIED

Table S3: Fully adjusted multivariate logistic regression coefficients with Z-codes. The dependent variable indicates if an HF patient has a 30-day all cause hospital readmission; n=586,929.

	coef	std err	z	P value
Intercept	-1.53	0.02	-64.56	0.00
C(Index HF Hospitalization Year, Ref('2019'))['2020']	-0.03	0.01	-3.99	0.00
C(Index HF Hospitalization Year, Ref('2019'))['2021']	-0.04	0.01	-5.26	0.00
C(Gender, Ref('Female'))['Male']	0.03	0.01	4.30	0.00
C(Region, Ref('NORTHEAST'))['MIDWEST']	0.13	0.01	10.40	0.00
C(Region, Ref('NORTHEAST'))['SOUTH']	-0.05	0.01	-4.56	0.00
C(Region, Ref('NORTHEAST'))['WEST']	-0.05	0.01	-4.92	0.00
C(Pay Type, Ref('COMMERCIAL'))['CASH']	-0.36	0.07	-5.46	0.00
C(Pay Type, Ref('COMMERCIAL'))['MEDICAID']	-0.03	0.02	-1.75	0.08
C(Pay Type, Ref('COMMERCIAL'))['MEDICARE']	0.01	0.01	1.99	0.05
C(Hospital Location, Ref('RURAL'))['URBAN']	-0.01	0.01	-0.38	0.71
C(Hospital Teaching Flag, Ref('No'))['Unknown']	0.07	0.01	6.16	0.00
C(Hospital Teaching Flag, Ref('No'))['Yes']	0.00	0.01	-0.45	0.65
C(Hospital Bed Size, Ref('500 or more beds'))['1-99 beds']	0.12	0.02	6.92	0.00
C(Hospital Bed Size, Ref('500 or more beds'))['100-199 beds']	0.14	0.01	13.20	0.00
C(Hospital Bed Size, Ref('500 or more beds'))['200-299 beds']	0.19	0.01	19.06	0.00
C(Hospital Bed Size, Ref('500 or more beds'))['300-499 beds']	0.16	0.01	18.37	0.00
Z55: Education and literacy	0.21	0.13	1.70	0.09
Z56: Employment	0.21	0.04	4.81	0.00
Z57: Occupational exposure risk	0.02	0.14	0.14	0.89
Z59: Housing and economic	0.37	0.02	19.02	0.00
Z60: Social environment	0.13	0.03	4.84	0.00
Z62: Upbringing	0.15	0.08	1.85	0.06
Z63: Primary support group	0.10	0.04	2.66	0.01
Z64-Z65: Psychosocial	0.09	0.06	1.56	0.12
Z77.1: Environmental pollution	0.21	0.16	1.36	0.18
Z91.89: Transportation	0.03	0.04	0.89	0.37
Age	-0.01	0.00	-20.30	0.00
CCI	0.08	0.00	77.00	0.00
1-year previous HF diagnosis (Yes)	-0.02	0.01	-2.58	0.01
1-year previous HF hospitalization (n =1)	0.11	0.01	8.96	0.00
1-year previous HF hospitalization (n >=2)	0.44	0.01	47.13	0.00

Table S4: Z55.x Education Literacy SDoH Odds Ratio of 30-day hospital readmission for heart failure

	**N (%)**	**Individual SDoH Subcodes Adjusted Odds Ratio**	**P Value**
Z55.0 Illiteracy and Low-Level Literacy	129 (40.8%)	1.30 (0.88, 1.91)	0.19
Z55.9 Education Literacy Unspecified	105 (33.2%)	1.75 (1.16, 2.62)	0.01
Z55.8 Other Problems Related to Education Literacy	77 (24.4%)	1.40 (0.86, 2.30)	0.18
Z55.3 Underachievement in School	15 (4.7%)	0.57 (0.13, 2.54)	0.46
Z55.1 Schooling Unavailable and Unattainable	2 (0.6%)	NA	NA
Z55.2 Failed School Examinations	2 (0.6%)	NA	NA
Z55.4 Educational Maladjustment Discord W Teachers Classmates	0 (0.0%)	NA	NA
Z55.5 Less Than a High School Diploma	0 (0.0%)	NA	NA

Table S5: Z62.xxx Upbringing SDoH Odds Ratio of 30-day hospital readmission for heart failure

	**N (%)**	**Individual SDoH Subcodes Adjusted Odds Ratio**	**P Value**
Z62.81x Personal History of Abuse in Childhood	603 (79.6%)	1.43 (1.20, 1.70)	0.00
Z62.82x Parent Child Conflict	78 (10.3%)	0.91 (0.54, 1.55)	0.73
Z62.2x Upbringing Away from Parents	47 (6.2%)	1.00 (0.49, 2.02)	1.00
Z62.89x Other Specified Problems Related to Upbringing	27 (3.6%)	1.20 (0.52, 2.77)	0.67
Z62.9 Problem Related to Upbringing Unspecified	11 (1.5%)	2.85 (0.86, 9.42)	0.09
Z62.0 Inadequate Parental Supervision and Control	1 (0.1%)	NA	NA
Z62.1 Parental Overprotection	(0.0%)	NA	NA
Z62.3 Hostility Towards and Scapegoating of Child	(0.0%)	NA	NA
Z62.6 Inappropriate Excessive Parental Pressure	(0.0%)	NA	NA

Table S6. Stepwise multivariate logistic regression coefficients with sub-Z-codes. The dependent variable indicates if an HF patient has a 30-day all cause hospital readmission; n=586,929.

	coef	std err	z	P value
Intercept	-1.54	0.02	-69.95	0.00
C(Index HF Hospitalization Year, Ref('2019'))['2020']	-0.03	0.01	-3.94	0.00
C(Index HF Hospitalization Year, Ref('2019'))['2021']	-0.04	0.01	-5.04	0.00
C(Gender, Ref('Female'))['Male']	0.03	0.01	3.99	0.00
C(Region, Ref('NORTHEAST'))['MIDWEST']	0.13	0.01	10.41	0.00
C(Region, Ref('NORTHEAST'))['SOUTH']	-0.05	0.01	-4.84	0.00
C(Region, Ref('NORTHEAST'))['WEST']	-0.05	0.01	-5.30	0.00
C(Hospital Teaching Flag, Ref('No'))['Unknown']	0.07	0.01	6.48	0.00
C(Hospital Teaching Flag, Ref('No'))['Yes']	0.00	0.01	-0.31	0.76
C(Pay Type, Ref('COMMERCIAL'))['CASH']	-0.35	0.07	-5.41	0.00
C(Pay Type, Ref('COMMERCIAL'))['MEDICAID']	-0.03	0.02	-1.90	0.06
C(Pay Type, Ref('COMMERCIAL'))['MEDICARE']	0.01	0.01	2.01	0.05
C(Hospital Bed Size, Ref('500 or more beds'))['1-99 beds']	0.12	0.02	6.88	0.00
C(Hospital Bed Size, Ref('500 or more beds'))['100-199 beds']	0.14	0.01	13.54	0.00
C(Hospital Bed Size, Ref('500 or more beds'))['200-299 beds']	0.19	0.01	19.54	0.00
C(Hospital Bed Size, Ref('500 or more beds'))['300-499 beds']	0.16	0.01	19.32	0.00
Age	-0.01	0.00	-20.15	0.00
CCI	0.08	0.00	77.22	0.00
1-year previous HF diagnosis (Yes)	-0.02	0.01	-2.52	0.01
1-year previous HF hospitalization (n =1)	0.11	0.01	8.90	0.00
1-year previous HF hospitalization (n >=2)	0.44	0.01	46.91	0.00
Z55.9 Education Literacy Unspecified	0.49	0.21	2.35	0.02
Z56.0 Unemployment Unspecified	0.20	0.04	4.55	0.00
Z56.9 Unspecified Problems Related to Employment	0.68	0.33	2.08	0.04
Z59.0 Homelessness	0.47	0.02	21.35	0.00
Z59.1 Inadequate Housing	0.22	0.16	1.44	0.15
Z59.4 Lack of Adequate Food	0.30	0.15	1.98	0.05
Z59.6 Low Income	-0.14	0.08	-1.75	0.08
Z59.8x Other Problems Related to Housing and Economic Circumstances	0.20	0.09	2.33	0.02
Z59.9 Problem Related to Housing and Economic Circumstances Unspecified	0.13	0.06	1.97	0.05
Z60.2 Problems Related to Living Alone	0.13	0.03	4.65	0.00
Z60.9 Problem Related to Social Environment Unspecified	0.25	0.12	2.13	0.03
Z62.81x Personal History of Abuse in Childhood	0.20	0.09	2.22	0.03
Z62.9 Problem Related to Upbringing Unspecified	0.94	0.62	1.53	0.13
Z63.1 Problems in Relationship with In-Laws	1.27	0.55	2.31	0.02
Z63.5 Disruption of Family by Separation and Divorce	0.29	0.11	2.70	0.01

Z63.7x Other Stressful Life Events Affecting Family and Household	0.21	0.12	1.76	0.08
